# Using the natural evolution of a rotavirus-specific human monoclonal antibody to predict the complex topography of a viral antigenic site

**DOI:** 10.1186/1745-7580-3-8

**Published:** 2007-09-18

**Authors:** Brett A McKinney, Nicole L Kallewaard, James E Crowe, Jens Meiler

**Affiliations:** 1 Department of Genetics, University of Alabama School of Medicine, 720 20^th ^Street South, Birmingham, 35294, USA; 2 Division of Infectious Diseases, Children's Hospital of Philadelphia, 34^th ^Street and Civic Center Boulevard, Philadelphia, 19104 USA; 3 Program in Vaccine Sciences, Departments of Microbiology and Immunology and Pediatrics, Vanderbilt University Medical Center, 21^st ^Avenue South and Garland Avenue, Nashville, 37232, USA; 4 Center for Structural Biology, Department of Chemistry, Vanderbilt University, 2201 West End Avenue, Nashville, 37232, USA

## Abstract

**Background:**

Understanding the interaction between viral proteins and neutralizing antibodies at atomic resolution is hindered by a lack of experimentally solved complexes. Progress in computational docking has led to the prediction of increasingly high-quality model antibody-antigen complexes. The accuracy of atomic-level docking predictions is improved when integrated with experimental information and expert knowledge.

**Methods:**

Binding affinity data associated with somatic mutations of a rotavirus-specific human adult antibody (RV6-26) are used to filter potential docking orientations of an antibody homology model with respect to the rotavirus VP6 crystal structure. The antibody structure is used to probe the VP6 trimer for candidate interface residues.

**Results:**

Three conformational epitopes are proposed. These epitopes are candidate antigenic regions for site-directed mutagenesis of VP6, which will help further elucidate antigenic function. A pseudo-atomic resolution RV6-26 antibody-VP6 complex is proposed consistent with current experimental information.

**Conclusion:**

The use of mutagenesis constraints in docking calculations allows for the identification of a small number of alternative arrangements of the antigen-antibody interface. The mutagenesis information from the natural evolution of a neutralizing antibody can be used to discriminate between residue-scale models and create distance constraints for atomic-resolution docking. The integration of binding affinity data or other information with computation may be an advantageous approach to assist peptide engineering or therapeutic antibody design.

## Background

The Rotavirus (RV) particle is composed of three concentric viral protein (VP) layers. The intermediate layer consists of VP6 (PDB accession code 1qhd [1]), which is the most antigenic RV protein in humans. RV is the most important viral cause of severe dehydrating diarrhea in infants and young children worldwide. Nearly all children will be infected with RV before three years of age regardless of social or economic status. Moreover, infant antibodies induced by virus exhibit poor functional activity compared to those of adults. We previously investigated the human antibody gene repertoire of RV-specific B cells from infected adults or infants. Although infant antibody gene sequences used the same immunodominant VH gene segments as adult sequences to respond to RV, there was a marked lack of somatic mutations in the infant antibody gene sequences [2-4].

Most recently we investigated the kinetic and functional advantage conferred by naturally occurring somatic mutations in VP6-specific human antibodies [5]. In this study, we investigated the effect of naturally occurring somatic mutations on the binding affinity of human antibodies to VP6. The effect of each somatic mutation in two highly mutated, naturally occurring adult Fabs (designated RV6-26 and RV6-25) was determined by mutating the amino acids one at a time back to the original germline sequence and measuring the resultant binding affinity. Our results suggested that the germline sequence codes for a low-affinity antibody for RV VP6, and somatic mutations in the HCDR2 region resulted in a higher-affinity adult antibody due to a much slower rate of dissociation. In the present study, our goal is to use data-guided computation to identify candidate VP6 residues for mutagenesis to further clarify the function of the naturally occurring mutations in RV6-26. We use a rational, integrative approach to identify candidate viral residues for mutagenesis to localize and define the complex surface topology of the major antigenic site on RV VP6.

Conformational peptides can be determined in detail from the atomic resolution structure of the antibody-antigen complex, which is most accurately characterized by X-ray crystallography. Due to difficulties often encountered in crystallizing complexes, other methods are needed to characterize the structure of many novel protein-protein complexes. Cryo-EM is a lower-resolution alternative when it is not feasible to determine the X-ray structure; specifically, when the complex has limited ability to form a crystal or it is difficult to produce sufficient quantities of the sample. While it is not possible to construct an atomic resolution structure with cryo-EM alone, the cryo-EM density can provide valuable insight and can act as a constraint for computational docking methods to predict an atomic-resolution structure. The antibody-antigen docking problem carried out in this paper is challenging due to the size of the VP6 trimer, which is composed of 1191 residues (397 for each monomer), and the potential flexibility of the antibody Fab, which is compose of 227 residues. However, biological knowledge helps to reduce the size of the docking search space. For example, it is known that the lower half of the VP6 is buried inside the RV double-layered particle and, thus, is not accessible to the antibody for binding. Docking predictions are most reliable when prior biological information is incorporated into the modeling process [6], and site-directed mutagenesis is a particularly useful source of biological information [7,8].

The diversity of antibodies is due to the six complementarity determining region (CDR) loops, whose flexibility and large number of surface accessible side chains allow the antibody to match a particular antigen epitope. When such induced conformational changes are large, one expects docking predictions to become less accurate if backbone flexibility is not incorporated. However, the large binding affinity of antibody-antigen associations in general, and the RV6-26-VP6 complex in particular, may limit the size of conformational shifts upon complex formation due to the evolutionary advantage of constrained loops for tighter binding [9,10]. We use the protein docking program RosettaDock [11] to perform simultaneous Monte-Carlo minimization of backbone displacement and backbone-dependent side-chain rotamer conformational changes.

RosettaDock has performed well in the blind Critical Assessment of Predicted Interactions (CAPRI) protein-protein docking challenge [12], including a situation in which one of the docking partners is a homology model with considerable structural errors [13]. In targets without significant backbone conformational changes, RosettaDock modeled nearly all interface side chains accurately while also finding nearly perfect rigid-body orientations of the partners. Although most RosettaDock predictions are performed without the use of prior biological information, recently an integrative strategy has been applied to computational docking of a homology model of an anti-tumor mAb with the known epitope of the epidermal growth factor receptor [8]. This filter strategy compared mutagenesis free energy changes computed by RosettaInterface [14] with experimental binding affinity information to predict a final structure with reasonable confidence.

In the current paper, our goal is to identify candidate VP6 epitopes of the human neutralizing antibody RV6-26 by defining an antibody orientation filter that combines the distances between interface residues of the docking partners with experimental binding affinity changes due to site-directed mutagenesis of somatic antibody mutations back to germline. Unique to this study is the use of somatic mutations, which occurred during the natural evolution of an adult human antibody. The orientation filter is used to select low-resolution RosettaDock model complexes for refinement. During a low-resolution RosettaDock search, each protein is represented as a backbone with the side chains approximated by their centroids. During a high-resolution search, all side-chain atoms are fully represented, and we use finer rotational and translational sampling as described below. After clustering the refined complexes, the RV6-26 antibody mutation that was most disruptive for binding following reversion to the germline sequence (Y66S [5]) acts as a probe for candidate VP6 contact residues.

## Methods

Since a crystal structure is not available for mAb RV6-26, we built a homology model of the structure using the Web Antibody Modeling (WAM) [15]. WAM uses a large number of known antibody structures as the knowledge database for homology modeling, and then applies *ab initio *molecular modeling for those parts of the antibody that are too variable for homology methods. For all docking runs, we included an alignment of our Fab with antibody binding subsequences of known antibody-antigen complexes, which allows RosettaDock to restrict the antibody from assuming an unlikely orientation of its CDR loops [11].

**Figure 1:**
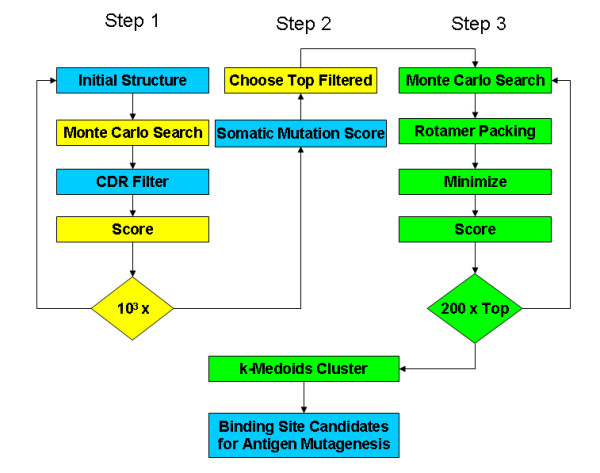
**Docking protocol**. Docking protocol with somatic mutation binding affinity score that integrates binding affinity data from antibody somatic mutations. Blue boxes indicate where prior biological information is integrated; yellow boxes indicate the use of low-resolution, residue-scale potentials; and green boxes indicate the use of high-resolution, atomic-scale potential functions.

Figure 1 illustrates the steps of the docking procedure. In the first step, we started with a complex obtained from a fit into a cryo-EM density with an approximate resolution of 22Å [5] and performed 1000 low-resolution Monte-Carlo simulations with RosettaDock, treating the antibody as a rigid body that diffuses toward the fixed VP6 trimer. In applications without a cryo-EM density to seed the simulations, it may be necessary to perform more than 1000 simulations. The starting structure was obtained by fitting the X-ray coordinates of the VP6 trimer into the three-dimensional cryo-EM reconstructions with the Situs suite of programs [16], and the X-ray coordinates of the RV6-26 model were fitted into the antibody portion of the density by visual inspection. Additionally, the CDR loops of the antibody were oriented toward the VP6. The low-resolution, residue-scale interaction potentials include residue-environment and residue-residue interaction terms derived from a database of interfaces, a contact score to reward contacting residues, a bump score to penalize overlapping residues, and an empirical score that rewards interface CDR residues that are known to make contact with antigens based on known antibody-antigen complexes.

**Figure 2:**
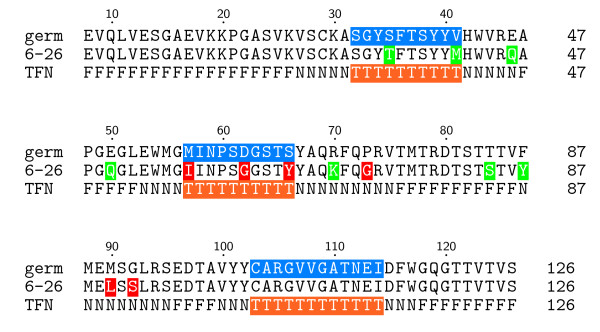
**Antibody binding affinity map**. Heavy-chain amino acid sequences for germline and RV6-26 adult antibodies, and the RosettaDock CDR scoring profile (TFN). Regions likely to be at the interface (CDRs) are highlighted in blue for the germline sequence. Active mutations in RV6-26, which result in improved binding with VP6, are highlighted in red, while neutral or negative mutations are highlighted in green. The TFN sequence is the RosettaDock scoring profile defined for this study that rewards True (T, orange) interface residues in the CDR region known to make contact with antigens, penalizes False (F) interface residues not observed to make antigen contact, and makes no contribution for Neutral (N) interface residues that rarely make contact with antigens and non-CDR active residues.

In the second step, we used a somatic mutation score (described below) based on binding affinity changes measured when naturally occurring RV6-26 antibody mutations were back-mutated to germline. This score was used to filter the 1000 low-resolution RosettaDock decoys. The motivation for using this data as a filter is that affinity is a measure of the evolutionary fitness of each mutation that occurs over the course of antibody evolution. The adult RV6-26 antibody contained 13 somatic mutations within the heavy chain. To identify which of these mutations affected binding to VP6, mutant antibodies were produced corresponding to the reversion of each somatic mutation amino acid back to the germline amino acid. Somatic mutations also occur in the light chain, but we focused on the 13 heavy chain mutations because the V_H_1–46 is the known immunodominant region. Ref. [5] provides experimental details of the measured binding affinity changes between each mutant and the wild-type RV6-26 antibody. Briefly, each mutant antibody was created, expressed, and purified and a detailed kinetic analysis performed using surface plasmon resonance. Most of the mutants retained binding equivalent to that of the wild-type Fab except for the six amino acids colored red in Fig. 2. For the somatic mutation filter in the current study, we discretized the equilibrium binding affinity changes for each mutation into active and neutral states (red and green, respectively, in Fig. 2).

Figure 2 shows the heavy chain amino acid sequences of the germline and adult RV6-26 antibodies, and summarizes the binding enhancement conferred by each amino acid. The numbering scheme used in Fig. 2 is derived from the immunoglobulin variable (V) gene database (VBASE), in which a unique antibody amino acid numbering system was introduced [17]. The first profile (germ) of the alignment in Fig. 2 shows the germline heavy-chain sequence, where the residues highlighted in blue are the CDR regions. The second profile (6–26) shows the somatic mutations of the RV6-26 antibody color coded in terms of their effect on VP6 binding. Amino acids highlighted in red were associated with enhanced antiviral activity of RV6-26, while amino acids highlighted in green had a neutral effect.

The third profile (TFN) of the alignment in Fig. 2 is the CDR scoring profile that is part of the low-resolution score in RosettaDock [11]. For our application, we defined True (T, orange) residues as CDR residues that are rewarded for being in the interface; False (F) residues as non-CDR residues that have not been observed to make antigen contact in known complexes and are penalized for being in the interface; and Neutral (N) interface residues as rarely occurring contact residues and non-CDR active residues, which make no contribution to the score. Even though the RV6-26 residues Gly73, Leu90, and Ser92 are not CDR residues, they were labeled as Neutral in the TFN profile because they were experimentally found to be active somatic mutations. This neutral labeling prevents the CDR score from excluding these non-CDR active somatic mutations from the interface.

For each complex, we created a matrix ***D ***of pair-wise distances between the C^α ^atoms of the 13 residue mutations of the RV6-26 antibody and a collection of VP6 residues from the interface, chosen based on visual inspection of the cryo-EM density. Virus interface residues were selected for the filter from two of the three VP6 chains: B-chain residues 197–213, 253–282, 287–301, 304, and 308; and C-chain residues 157–173, 236–247, and 351–374. The rows of the matrix ***D ***correspond to theVP6 residues at the interface and the columns to the antibody mutations highlighted in Fig. 2. For a given complex, we can determine the shortest distance of each antibody mutation to the VP6 interface from the matrix ***D***. These distances yield a vector ***d ***whose elements are given by

di=min⁡jDij
 MathType@MTEF@5@5@+=feaafiart1ev1aaatCvAUfKttLearuWrP9MDH5MBPbIqV92AaeXatLxBI9gBaebbnrfifHhDYfgasaacH8akY=wiFfYdH8Gipec8Eeeu0xXdbba9frFj0=OqFfea0dXdd9vqai=hGuQ8kuc9pgc9s8qqaq=dirpe0xb9q8qiLsFr0=vr0=vr0dc8meaabaqaciaacaGaaeqabaqabeGadaaakeaacqWGKbazdaWgaaWcbaGaemyAaKgabeaakiabg2da9maaxababaGagiyBa0MaeiyAaKMaeiOBa4galeaacqWGQbGAaeqaaOGaemiraq0aaSbaaSqaaiabdMgaPjabdQgaQbqabaaaaa@3A4B@

whose length is equal to the number of antibody mutations.

The filter score *F *of a complex is given by the following sum over all antibody mutations *M *(*M = 13 *for RV6-26)

F=∑i=1Mfdc(di)
 MathType@MTEF@5@5@+=feaafiart1ev1aaatCvAUfKttLearuWrP9MDH5MBPbIqV92AaeXatLxBI9gBaebbnrfifHhDYfgasaacH8akY=wiFfYdH8Gipec8Eeeu0xXdbba9frFj0=OqFfea0dXdd9vqai=hGuQ8kuc9pgc9s8qqaq=dirpe0xb9q8qiLsFr0=vr0=vr0dc8meaabaqaciaacaGaaeqabaqabeGadaaakeaacqWGgbGrcqGH9aqpdaaeWbqaaiabdAgaMnaaBaaaleaacqWGKbazdaWgaaadbaGaem4yamgabeaaaSqabaGccqGGOaakcqWGKbazdaWgaaWcbaGaemyAaKgabeaakiabcMcaPaWcbaGaemyAaKMaeyypa0JaeGymaedabaGaemyta0eaniabggHiLdaaaa@3E74@

where the contribution from each residue is

fdc(di)={1,di≤dc∧i∈active(3a)12,di≥dc∧i∈neutral(3b)0,otherwise(3c)
 MathType@MTEF@5@5@+=feaafiart1ev1aaatCvAUfKttLearuWrP9MDH5MBPbIqV92AaeXatLxBI9gBaebbnrfifHhDYfgasaacH8akY=wiFfYdH8Gipec8Eeeu0xXdbba9frFj0=OqFfea0dXdd9vqai=hGuQ8kuc9pgc9s8qqaq=dirpe0xb9q8qiLsFr0=vr0=vr0dc8meaabaqaciaacaGaaeqabaqabeGadaaakeaacqWGMbGzdaWgaaWcbaGaemizaq2aaSbaaWqaaiabdogaJbqabaaaleqaaOGaeiikaGIaemizaq2aaSbaaSqaaiabdMgaPbqabaGccqGGPaqkcqGH9aqpdaGabeqaauaabeqadmaaaeaacqaIXaqmaeaacqGGSaalaeaacqWGKbazdaWgaaWcbaGaemyAaKgabeaakiabgsMiJkabdsgaKnaaBaaaleaacqWGJbWyaeqaaOGaey4jIKTaemyAaKMaeyicI4mcbeGae8xyaeMae83yamMae8hDaqNae8xAaKMae8NDayNae8xzauMae8hiaaIae8hkaGIae83mamJae8xyaeMae8xkaKcabaWaaSGaaeaacqaIXaqmaeaacqaIYaGmaaaabaGaeiilaWcabaGaemizaq2aaSbaaSqaaiabdMgaPbqabaGccqGHLjYScqWGKbazdaWgaaWcbaGaem4yamgabeaakiabgEIizlabdMgaPjabgIGiolab=5gaUjab=vgaLjab=vha1jab=rha0jab=jhaYjab=fgaHjab=XgaSjab=bcaGiab=HcaOiab=ndaZiab=jgaIjab=LcaPaqaaiab=bdaWaqaaiabcYcaSaqaaiab=9gaVjab=rha0jab=HgaOjab=vgaLjab=jhaYjab=Dha3jab=LgaPjab=nhaZjab=vgaLjab=bcaGiab=HcaOiab=ndaZiab=ngaJjab=LcaPaaaaiaawUhaaaaa@8252@

and *d*_*c *_is a distance cutoff in Angstroms that characterizes our measure of closeness between the antibody and antigen. We classify a somatic mutation as "active" if back-mutation to germline has a disruptive effect on binding to the antigen. "Neutral" somatic mutations are non-disruptive when mutated back to germline. In Eq. (3a), an active somatic mutation contributes 1 to the affinity filter score of a complex if this residue is within *d*_*c *_Angstroms of the antigen interface. In our application, we chose a relatively loose cutoff of *d*_*c*_*= 12*Å to allow all active mutations the possibility of contributing to the score, including ones that may be more distant from the interface. It has been observed that many affinity-maturing mutations in singe chain Fv antibodies correspond to residues that are more distant from the interface [18,19]. A smaller cutoff would exclude the contribution to the score of residues that are involved in affinity maturation yet may not make direct contact with the antigen. In Eq. (3b), we allow a neutral or negative somatic mutation to contribute 1/2 to the score if it is more distant from the interface than *d*_*c*_. This essentially penalizes neutral somatic mutations that are closer to the interface than the cutoff distance. Of course, it is still possible for non-disruptive mutations to be near the interface, so this soft distance constraint penalizes but does not exclude neutral residues from contacting the antigen. In the final piece (Eq. 3c), all other somatic mutations do not contribute to the filter score of Eq. (2).

In the third step of Fig. 1, we performed a high-resolution docking refinement of the top filtered complexes. A backbone-dependent rotamer packing algorithm is used for side-chain repacking [20]. From each of the best low-resolution complexes ranked by filter score, we created 200 high-resolution decoys using the perturbation triplet (2Å, 2Å, 20°). This perturbation triplet represents a search volume with respect to the line connecting the protein centers. The first number refers to translation along the line, the second refers to translation in the plane perpendicular to the line, and the third refers both to rotation around the axis defined by the line and tilt relative to the axis.

The resulting high-resolution decoys were ranked according to their full-atom scores. Candidate complexes were determined by an additional round of refinement (1Å, 1Å, 10°) following k-medoids (k = 3) clustering of the top Rosetta-scoring complexes. Root-mean-square deviation (RMSD) of the alpha-carbon coordinates was used as the cluster metric, and the cluster location was given by the decoy with the lowest full-atom energy score within the cluster. We used k-medoids clustering because, unlike hierarchical clustering, it does not require linkage assumptions, and it is simpler than mixture model clustering. Unlike model-based clustering, which has the advantage of a statistical model that allows it to estimate the number of clusters, we must *a priori *choose the number of clusters in k-medoids; however, this choice is easily validated by visual inspection of the 3D complexes. The candidate binding sites were determined from the final candidate complexes by finding the VP6 residues within a 5Å radius of the RV6-26 Tyr66. This mutation was chosen as a computational probe because it had the largest effect on binding affinity as back-mutation of this residue resulted in an 83-fold decreased rate of dissociation [5].

## Results

**Figure 3:**
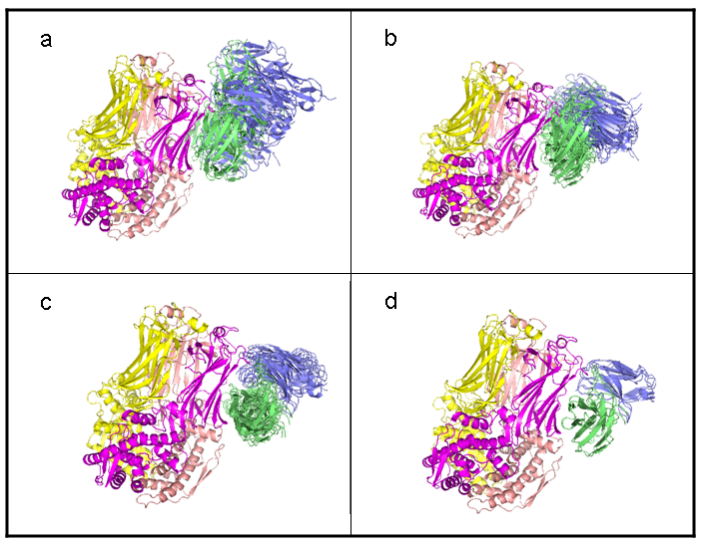
**Affinity filter clusters**. Top low-resolution complexes clustered according to filter score (FS). (a) FS = 7.5, (b) FS = 8.0, (c) FS = 8.5, (d) FS = 9.0. The RV6-26 Fv chains are shaded blue (light chain) and green (heavy chain). The RMSD of the clusters tightens as the score improves (increases).

Following the creation of 1000 antibody-antigen decoy complexes with the fit into the cryo-EM density map in Ref. [5] as a starting structure, we calculated the somatic mutation affinity score (Eq. 2) of each complex for the filter in step 2 of Fig. 1. Because six somatic mutations were labelled active and seven as neutral, the maximum affinity filter score is 9.5. The highest score of the low-resolution complexes was 9.0. Figure 3 shows the top low-resolution RV6-26-VP6 complexes clustered by affinity filter score. As the filter score of the complexes increases, the RMSD tightens between complexes within the same cluster. This suggests that the level of uncertainty in the location of the binding partners decreases with increasing somatic mutation affinity score.

**Figure 4:**
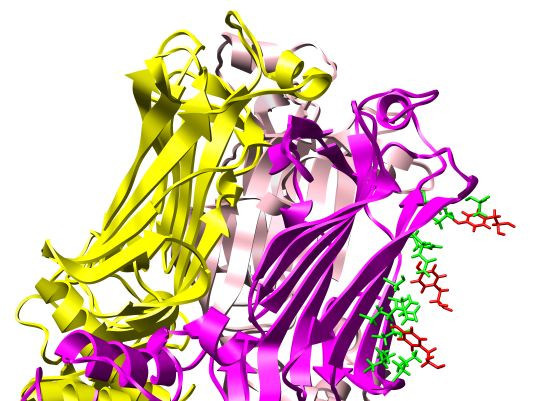
**Candidate binding sites**. Candidate residues involved in antibody-antigen (RV6-26-VP6) binding as predicted from the three best computational docking refinements. The antibodies for the three complexes are hidden except for the tyrosines (red), which act as probes for detecting candidate viral residues for mutagenesis (green). VP6 side chains (all in the same monomer) that are within 5Å of a tyrosine probe are shown in green.

Starting from the best 45 filtered low-resolution complexes from the top four clusters in Fig. 3, we created 200 high-resolution decoys each. The resulting 9000 high-resolution decoys were ranked according to their energy scores. We created the 9000 high-resolution decoy complexes by searching a small region of space defined by (2Å, 2Å, 20°) around each low-resolution starting complex. We then ranked the high-resolution decoys by full-atom score and k = 3 medoid clustered the top 100 with a pair-wise RMSD metric. The location of two of the three clusters corresponded to the top two full-atom scores, and the location of the third cluster corresponded to the fourth full-atom ranked structure. This third cluster had very close RMSD with one of the other two clusters, so we chose the top two complexes for a final refinement step (200 decoys searched in a volume defined by 1Å, 1Å, and 10°). The best refinements of these two clusters, along with a repacked and minimized structure of the best-scoring (*F = 9.0*) low-resolution complex in Fig. 3, represent the three candidate modes of binding summarized in Fig. 4.

In Fig. 4, the VP6 residues shaded green are within a 5Å radius of the RV6-26 Tyr66 of each candidate mode of binding. For clarity, only the Tyr66 residues are represented from the antibodies. These tyrosines in red act as computational probes of VP6 residues. Our approach has the effect of marching these probes up the outer sheet of the neck of one of the VP6 monomers. The probes in Fig. 4 allow us to propose VP6 residues for mutation and measurement of binding affinity changes to discriminate between the candidate modes of binding. The antibody probes suggest the following VP6 residues for mutation and binding affinity measurement to test whether they contribute to the antigenic site that is the target for binding by the RV6-26 antibody: Glu262, Leu264, Gln268, Ile269, Gln274, Arg289, Met295, Arg296, Pro297, and Pro298.

**Figure 5:**
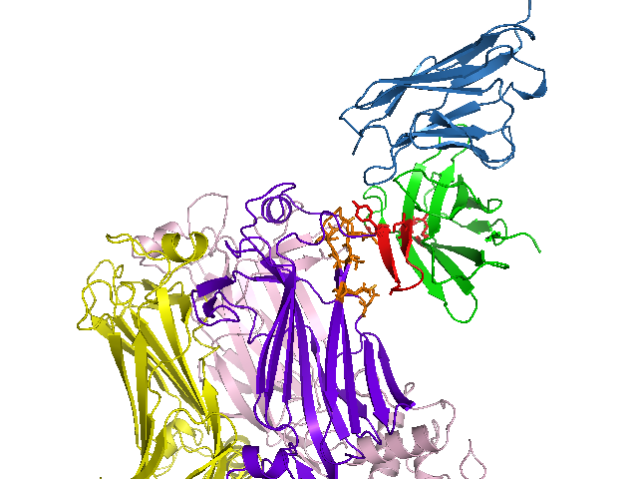
**Proposed high-resolution structure of antibody-antigen complex**. Best-scoring high-resolution (RV6-26-VP6) complex. The heavy chain of the RV6-26 Fab is shaded green and the light chain blue. Interface VP6 residues on chain B within 5Å of the active antibody residues are shaded orange. The active Fab residues that most affect binding affinity upon back-mutation have side chains shaded red. The CDR2 loop, which contains half of the active residues, is also shaded red.

**Figure 6:**
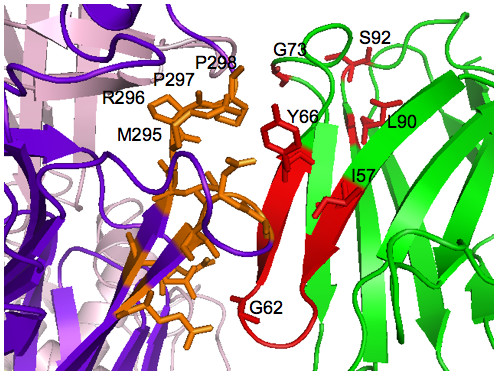
**Magnification of proposed high-resolution antibody-antigen complex**. Magnified 3D atomic structure of Fig. 5 complex centered on the candidate interface. The light chain is hidden, which otherwise obscures the interface. Active heavy chain somatic mutations and VP6 virus residues that make contact with the Y66 antibody residue are labelled.

The best scoring high-resolution model, shown in Fig. 5 and magnified in Fig. 6, is proposed as the Ab-Ag complex most consistent with current experimental information. The complex in Fig. 5 corresponds to the uppermost tyrosine along the VP6 neck in Fig. 4. The Model in Fig. 5 and 6 suggests that the epitope residues nearest the Tyr66 antibody residue are Met295, Arg296, Pro297, and Pro298. These and the other proposed conformational epitope residues are shaded orange. In Figs. 5 and 6, four of the six active antibody residues are within 5Å of the interface; namely, Ile57, Gly62, and Tyr66 from CDR2 (see also Fig. 2) and Gly73. All six active antibody residues are within 12Å of the interface.

## Discussion

In this study, we proposed an integrative structure-based computational approach to identify the RV VP6 epitope for a human adult antibody. Successful computational docking strategies have been developed that integrate experimental data, such as NMR chemical shift perturbations, residual dipolar couplings, and mutagenesis data [6]. In these strategies, experimental information typically is used either as a filter to validate docked complexes or a wrapper to restrain potential complexes during sampling. For example, HADDOCK incorporates biologic information as an additional energy term to be minimized during sampling [21,7]. To increase the power to identify the correct binding site, the strategy used in the current paper integrated knowledge-based restraints into the modeling process before, during and after docking; using, respectively, a cryo-EM density, CDR alignment score, and binding affinity data for somatic mutations. The cryo-EM density was used to constrain the starting point for docking simulations, and the CDR information was used to restrict unrealistic antibody orientations during docking runs. We combined experimental binding affinity changes, which quantify the evolutionary fitness of each mutation, with soft distance constraints estimated from the cryo-EM. We integrated this information into a score for each complex that acts as a post-processing filter after the docking models were created. The best, filtered complexes underwent high-resolution refinement then clustering to give three candidate binding modes.

This strategy is unique in its use of naturally occurring somatic mutations of a human antibody as a filter. The fact that all six active antibody residues were within 12Å of the interface (Fig. 5) corroborates the self-consistency of our choice of distance cutoff in the somatic mutation score (Eq. 2). A loose distance restraint was chosen due to previous observations that active residues often do not make direct contact with antigen [18,19]. The somatic mutation score was used as a post-docking complex filter not unlike the filter used in Ref. [8] in which computational and experimental mutagenesis results were compared. In this study, we used RosettaDock, but the somatic filter score could be used with other docking programs. Another strategy worth pursuing is to use the somatic mutation score as a wrapper as opposed to a filter, which would involve wrapping the somatic mutation score into the energy function during sampling to obtain a collection of complexes enriched for active residues in the interface. A simple way to implement a wrapper with RosettaDock would be to use the CDR alignment profile.

In the applied result in this paper, the algorithm had the advantage of an initial distance constraint provided by a cryo-EM density. This gave us added confidence in the antigenic-site candidates found by the algorithm, and it provided a starting point for validation of the algorithm on complexes where only binding affinity data is known. Unlike the two candidate complexes (whose antibody tyrosines were further down the VP6 neck in Fig. 4), the final complex in Fig. 5 is entirely within the cryo-EM density map (data not shown). However, to avoid undue bias from the cryo-EM, we retain the other, less conservative complex predictions as candidate mutagenesis sites on the VP6 to validate in a subsequent study. If a cryo-EM density were not available, a reasonable strategy would be to simulate a large (10^5^) number of low-resolution complexes to explore the global space of potential interfaces, and then choose an encompassing region around the largest cluster as the VP6 residues near the interface.

Affinity changes measured for the reversion of somatic mutations provides information about interface residues but not about specific antigen contacts. Additional feedback with experiment is necessary to unambiguously identify the interface. Thus, binding affinity changes caused by directed mutagenesis of the proposed viral protein residues will be used to eliminate false positive epitopes that we suspect in the less conservative complexes. Once residues from the epitope have been identified through this process, binding affinity changes from VP6 mutagenesis will be used together with the naturally evolved somatic mutations of the RV6-26 antibody that enhance binding affinity (Fig. 2) to infer distance constraints for a more accurate prediction of the complex than Fig. 5. The resolution of our final model in Fig. 5 is diminished by the potential error in the antibody homology model. After we obtain distance constraints from the VP6 mutagenesis follow-up study, we will perform conformational sampling of the antibody loop, constrained at the active site, to account for errors in the antibody homology model and loop conformational changes that may occur upon binding.

In addition to conformational epitope identification, computational docking that integrates experimental data may lead to more general *in silico *procedures for the prediction of antibody mutations that produce higher affinity, higher specificity binding to a desired target macromolecule than the natural antibody sequence. Studies involving the *in vitro *evolution of antibodies have shown that the diversity of structurally stable antibody sequences is much greater than the diversity observed in nature, which suggests that this diversity could be exploited for therapeutic antibody design [22,23]. Computation coupled with feedback to experimental information is a promising *in silico-in vitro *integrative approach to guide the design or enhancement of therapeutic antibodies.

## Competing interests

The authors declare that they have no competing interests.

## Authors' contributions

BAM and JM conceived of the computational strategy. BAM carried out the computational analysis and drafted the manuscript. NLK carried out the experimental studies, and JEC oversaw the experimental design. All authors contributed to the draft of the manuscript and helped interpret the results. All authors read and approved the final manuscript.

## References

[B1] Mathieu M, Petitpas I, Navaza J, Lepault J, Kohli E, Pothier P, Prasad BV, Cohen J, Rey FA (2001). Atomic structure of the major capsid protein of rotavirus: implications for the architecture of the virion. Embo J.

[B2] Weitkamp JH, Kallewaard N, Kusuhara K, Bures E, Williams JV, LaFleur B, Greenberg HB, Crowe JE (2003). Infant and adult human B cell responses to rotavirus share common immunodominant variable gene repertoires. J Immunol.

[B3] Weitkamp JH, Kallewaard N, Kusuhara K, Feigelstock D, Feng N, Greenberg HB, Crowe JE (2003). Generation of recombinant human monoclonal antibodies to rotavirus from single antigen-specific B cells selected with fluorescent virus-like particles. J Immunol Methods.

[B4] Weitkamp JH, Lafleur BJ, Greenberg HB, Crowe JE (2005). Natural evolution of a human virus-specific antibody gene repertoire by somatic hypermutation requires both hotspot-directed and randomly-directed processes. Hum Immunol.

[B5] Kallewaard NL, McKinney BA, Gu Y, Chen A, Prasad BVV, Crowe JE (2007). Functional Maturation of the Human Antibody Response to Rotavirus.

[B6] van Dijk AD, Boelens R, Bonvin AM (2005). Data-driven docking for the study of biomolecular complexes. Febs J.

[B7] Norledge BV, Petrovan RJ, Ruf W, Olson AJ (2003). The tissue factor/factor VIIa/factor Xa complex: a model built by docking and site-directed mutagenesis. Proteins.

[B8] Sivasubramanian A, Chao G, Pressler HM, Wittrup KD, Gray JJ (2006). Structural model of the mAb 806-EGFR complex using computational docking followed by computational and experimental mutagenesis. Structure.

[B9] Betts MJ, Sternberg MJ (1999). An analysis of conformational changes on protein-protein association: implications for predictive docking. Protein Eng.

[B10] Thorpe IF, Brooks CL (2007). Molecular evolution of affinity and flexibility in the immune system. Proc Natl Acad Sci USA.

[B11] Gray JJ, Moughon S, Wang C, Schueler-Furman O, Kuhlman B, Rohl CA, Baker D (2003). Protein-protein docking with simultaneous optimization of rigid-body displacement and side-chain conformations. J Mol Biol.

[B12] Schueler-Furman O, Wang C, Baker D (2005). Progress in protein-protein docking: atomic resolution predictions in the CAPRI experiment using RosettaDock with an improved treatment of side-chain flexibility. Proteins.

[B13] Daily MD, Masica D, Sivasubramanian A, Somarouthu S, Gray JJ (2005). CAPRI rounds 3–5 reveal promising successes and future challenges for RosettaDock. Proteins.

[B14] Kortemme T, Baker D (2002). A simple physical model for binding energy hot spots in protein-protein complexes. Proc Natl Acad Sci USA.

[B15] Whitelegg NR, Rees AR (2000). WAM: an improved algorithm for modelling antibodies on the WEB. Protein Eng.

[B16] Wriggers W, Milligan RA, McCammon JA (1999). Situs: A package for docking crystal structures into low-resolution maps from electron microscopy. J Struct Biol.

[B17] Lefranc MP (2004). IMGT, The International ImMunoGeneTics Information System. Methods Mol Biol.

[B18] Daugherty PS, Chen G, Iverson BL, Georgiou G (2000). Quantitative analysis of the effect of the mutation frequency on the affinity maturation of single chain Fv antibodies. Proc Natl Acad Sci USA.

[B19] Ramirez-Benitez MC, Almagro JC (2001). Analysis of antibodies of known structure suggests a lack of correspondence between the residues in contact with the antigen and those modified by somatic hypermutation. Proteins.

[B20] Dunbrack RL, Cohen FE (1997). Bayesian statistical analysis of protein side-chain rotamer preferences. Protein Sci.

[B21] Dominguez C, Boelens R, Bonvin AM (2003). HADDOCK: a protein-protein docking approach based on biochemical or biophysical information. J Am Chem Soc.

[B22] Bond CJ, Wiesmann C, Marsters JC, Sidhu SS (2005). A structure-based database of antibody variable domain diversity. J Mol Biol.

[B23] Sidhu SS, Fellouse FA (2006). Synthetic therapeutic antibodies. Nat Chem Biol.

